# SuFEx-enabled high-throughput medicinal chemistry for developing potent tamoxifen analogs as Ebola virus entry inhibitors

**DOI:** 10.3389/fimmu.2025.1533037

**Published:** 2025-04-28

**Authors:** Lucas Dada, Emiko Nagai, Sashank Agrawal, Ariel S. Wirchnianski, Ian A. Wilson, Kartik Chandran, Seiya Kitamura

**Affiliations:** ^1^ Department of Biochemistry, Albert Einstein College of Medicine, Bronx, NY, United States; ^2^ Department of Integrative Structural and Computational Biology, The Scripps Research Institute, La Jolla, CA, United States; ^3^ Department of Microbiology & Immunology, Albert Einstein College of Medicine, Bronx, NY, United States; ^4^ Skaggs Institute for Chemical Biology, The Scripps Research Institute, La Jolla, CA, United States

**Keywords:** Ebola, small molecule antiviral drugs, drug discovery, SuFEx, direct-to-biology

## Abstract

Ebola virus (EBOV) causes severe hemorrhagic fever with a high mortality rate in humans. In acute infection, an abnormal immune response results in excessive inflammatory cytokines and uncontrolled systemic inflammation that can result in organ damage and multi-organ failure. While vaccines and monoclonal antibody therapies are available, there is an urgent need for effective small-molecule antivirals against EBOV. Here, we report on the optimization of tamoxifen, an EBOV-glycoprotein (GP) binder that inhibits viral entry, using our Sulfur-Fluoride Exchange (SuFEx) click chemistry-based high-throughput medicinal chemistry (HTMC) strategy. Using a “Direct-to-Biology” approach, we generated a focused library of 2,496 tamoxifen analogs overnight and screened them in a cell-based pseudo-EBOV infection assay. The HTMC workflow enabled the development of a potent EBOV entry inhibitor with submicromolar EC_50_ cellular antiviral activity and more than 50-fold improvement in binding affinity against EBOV-GP compared to the parent compound. Our findings underscore the use of SuFEx-enabled HTMC for rapidly generating and assessing potential therapeutic candidates against viral and immune-mediated diseases in a cell-based assay.

## 1 Introduction

Ebola virus (EBOV) is an enveloped, single-stranded, negative-sense RNA virus in the family *Filoviridae* (1). EBOV infection in humans can lead to Ebola virus disease (EVD), a clinical syndrome initially characterized by nonspecific symptoms, which later progress to severe gastrointestinal issues and hemorrhagic complications with a lethality rate as high as 90% (2–4). While other orthoebolaviruses, such as Sudan virus and Marburg virus, can also cause human disease with substantial mortality, EBOV has been responsible for the majority of recorded human outbreaks and therefore, remains to be of considerable public health concern (5). The unprecedented 2014–2016 EVD epidemic in West Africa, as well as the 2022 outbreak in the Democratic Republic of the Congo have underscored the potential of EVD to trigger severe health emergencies on a regional scale (6, 7).

EBOV causes an acute and serious viral hemorrhagic fever disease, which is often fatal if left untreated. EBOV primarily targets host macrophages leading to cell activation and systemic cytokine storm. Fatal infection is associated with an inhibited interferon response and lymphopenia. Cytokine storms are a hallmark of EVD and play a central role in its pathogenesis, marked by the induction of both pro- and anti-inflammatory responses (8, 9). Despite the high mortality rate associated with EVD, some patients survive and, in certain cases, develop chronic manifestations that may resemble inflammatory or autoimmune conditions (10). It has been demonstrated that this EBOV-induced autoimmunity is involved in post EVD syndrome (11). These features of EVD highlight the necessity for effective therapeutic approaches against EBOV. Recently, EBOV vaccine and monoclonal antibody-based therapeutics have been approved by the FDA (12, 13). However, there are currently no FDA-approved small-molecule drugs with demonstrated efficacy against filovirus infection and/or disease in humans, despite the increasing frequency with which these viruses are causing outbreaks of global concern (14). Therefore, there is a pressing and unmet need for effective therapies to prevent EBOV entry and subsequent infection.

In the pursuit of drug discovery against EVD, several high-throughput screening campaigns have been conducted that yielded hit compounds (15, 16). However, only limited medicinal chemistry optimization and *in vivo* follow-up studies were performed previously, partially due to the iterative cycle of medicinal chemistry process being time-consuming and labor-intensive. Our group has developed the first-of-its-kind high-throughput medicinal chemistry (HTMC) platform using click chemistry reactions, in particular Sulfur-Fluoride Exchange (SuFEx) reactions (17–21), to accelerate the medicinal chemistry process. The unanticipated discovery that iminosulfur oxydifluoride (difluoride, RN=S(O)F_2_)-containing molecules react overnight with amines to yield products with >80% conversion in a biocompatible condition (22) allowed us to perform large-scale structure-activity relationship (SAR) studies directly from reaction mixtures. We have used this new type of click chemistry reaction to rapidly synthesize focused libraries of lead compound analogs in a miniaturized format, directly assess the products with biological assays (also known as “Direct-to-Biology (D2B)” approach) (23), and develop drug-like ligands with improved biological potency. We have previously demonstrated the utility of our SuFEx-based HTMC method to improve the potency and specificity of chemical probes against a bacterial pathogenic protease, a human leukemia-associated transcriptional coactivator, the influenza hemagglutinin stem, and molecular glues (17–21).

In this study, we applied our SuFEx-based HTMC platform to an inhibitor of the EBOV-glycoprotein (GP) for the expedited analysis of structure-activity relationships. Tamoxifen, an EBOV-GP inhibitor (24, 25), was used as a starting scaffold for diversification because of its suitability for the synthetic preparation of a SuFExable analog. With the support of an automated liquid handling robot, a focused library of 2,496 compound analogs was synthesized in a single batch and screened using a cell-based EBOV entry assay. This study provides a large-scale SAR dataset of tamoxifen analogs as EBOV entry inhibitors, which will be valuable for the further development of small molecule therapeutics against EVD. Importantly, the HTMC workflow enabled the discovery of an analog with a 50-fold improved binding affinity compared to tamoxifen. Our study showcases the successful application of the SuFEx-based HTMC platform for the accelerated structure-activity relationship study of small molecule inhibitors, ultimately providing next-generation therapeutic modalities against viral infection and autoimmune diseases.

## 2 Materials and methods

### 2.1 Cells and viruses

The Vero African green monkey kidney cells in this study were obtained from American Type Culture Collection (ATCC), Cat# CCL-81. Vero cells were cultured in Dulbecco’s modified Eagle medium (DMEM, Corning, NY) containing 10% fetal bovine serum (FBS, Phoenix Scientific, St. Joseph, MO), 1% penicillin-streptomycin (Corning, NY), and GlutaMax (Thermo Fisher Scientific, Waltham, MA). Cells were maintained at 37°C in 5% CO_2_. Generation and propagation of recombinant vesicular stomatitis virus (rVSV) encoding enhanced green fluorescent protein (eGFP) in the first position and replacing VSV G with the EBOV-GP (EBOV/H.sap-tc/COD/76/Yambuku-Mayinga) or Lassa virus (LASV)-GP (Josiah) were previously described (26, 27).

### 2.2 Computational protocol

Docking studies were carried out using Glide-SP (Schrödinger modeling suite, versions 2024-1 to 2024-3) to obtain the binding mode for tamoxifen into the proposed binding site. Standard option joint with the SP algorithm was applied for pose generation and evaluation. The X-ray structure for the Zaire EBOV-GP in complex with toremifene (PDB: 5JQ7) was used as a template for the docking.

### 2.3 Chemical synthesis

The details of chemical synthesis and characterization are described in **Supplementary Material**. The SuFEx-based library synthesis was adapted from a method described previously (17) in 384-well plate format. Briefly, to a DMSO solution of the iminosulfur oxydifluoride derivative **1** was added amine library in DMSO and sodium phosphate buffer (pH 9.0, 0.2 M) subsequently. The compounds were synthesized with difluoride concentration at 1 mM and a solvent mixture DMSO:buffer 3:1. The reaction mixtures were shaken at room temperature overnight and then used directly for activity measurement with 5000-fold dilution.

### 2.4 Screening and EC_50_ measurement using an EBOV-GP-pseudotyped virus

The virus was titrated to achieve an infection rate of approximately 50%. Vero cells were seeded at a density of 2.0 × 10^4^ cells/well in a 384-well plate and incubated for 24 hours at 37°C in 5% CO_2_. The cell culture medium was then replaced with 20 μL/well of virus culture medium (DMEM containing 2% FBS, 1% penicillin-streptomycin, and GlutaMax), and 0.2 μL/well of the compound library in DMSO was added using the Bravo Automated Liquid Handling Platform (Agilent Technologies, Santa Clara, CA). Subsequently, 20 μL/well of virus solution was added, and the cells were incubated for 16 hours at 37°C in 5% CO_2_. Infected cells were stained with Hoechst (Thermo Fisher Scientific, Waltham, MA) to visualize nuclei and fixed with 4% paraformaldehyde. Infectivity of VSV pseudotype was measured by automated enumeration of eGFP^+^ cells using a Cytation 5 reader (BioTek, Winooski, VT), as previously described (26). Quantification was done using Gen5 data analysis software (BioTek, Winooski, VT). For each plate, DMSO-treated infected and non-infected control wells were included to calculate the inhibition rate as: Inhibition rate (%) = (Infection control - Sample)/(Infection control - Non-infection control) × 100.

Dose-response assays were performed under the same experimental conditions as the library screening. Compounds were prepared in two-fold serial dilutions in DMSO and 0.2 μL/well was added to the cell culture media using the Bravo Automated Liquid Handling Platform. The EC_50_ values were calculated using GraphPad Prism 10.

### 2.5 Cytotoxicity measurement

Cells were seeded under the same conditions as the antiviral assay. The cell culture medium was replaced with 40 μL/well of virus infection medium without virus, and compound solutions prepared as two-fold serial dilutions were added at 0.2 μL/well using the Bravo platform. After 16 hours, Promega^®^ CellTiter-Glo^®^ 2.0 (Promega, Madison, WI) was added, and cell viability was assessed following the manufacturer’s instruction. The CC_50_ values were calculated using GraphPad Prism 10.

### 2.6 Expression and purification of EBOV-GP trimer protein

The gene fragment encoding the extracellular domain of the Zaire EBOV (strain Mayinga-76) glycoprotein (UniProt ID: KB-Q05320) was synthesized as described previously (28). This gene was inserted into the mammalian expression vector pHCMV3, with an Ig Cκ leader sequence at the N-terminus to enable secretion. A foldon trimerization domain from bacteriophage T4 fibritin was added to the C-terminus to promote trimerization, along with a 6×His tag for affinity purification.

The plasmid was transfected into human Expi293S cells to produce the EBOV-GP trimers. After six days of incubation, the culture medium containing the secreted trimers was collected. The protein was purified using Ni-NTA affinity chromatography with Ni Sepharose Excel resin and dialyzed overnight into 1× PBS. The sample was concentrated and further purified via size-exclusion chromatography on a Superdex 200 HiLoad 16/600 column pre-equilibrated with 1× TBS. The protein peak corresponding to the EBOV-GP trimers was identified, collected, and concentrated to a final concentration of 2 mg/mL.

### 2.7 Microscale thermophoresis (MST)

Recombinant EBOV-GP protein was labeled using the Monolith Protein Labeling Kit RED-tris-NTA 2nd Generation dye (Cat #MO-L018, NanoTemper Technologies, Germany) following the manufacturer’s instructions. Specifically, 125 nM EBOV-GP was incubated with 25 nM dye in 25 mM HEPES pH 7.5, 100 mM NaCl, 0.005% tween-20 in the dark at room temperature for 30 min. The sample was centrifuged for 10 min at 4°C and 14,000 g and the supernatant was transferred to a fresh tube. To determine the *K*_D_ of EBOV-GP to tamoxifen and its analogs, 125 nM labeled EBOV-GP was incubated with increasing concentrations of small molecules in the same buffer with 1% DMSO. Samples were loaded into standard glass capillaries (Monolith NT.155 Capillaries) and analyzed by MST using a Monolith NT.115 Blue/Red, LED power and IR laser power of 60%. Samples showed no aggregation according to post-run analysis using the Monolith data collection software (NanoTemper). Fraction bound and error were generated by NanoTemper software (MO.Affinity Analysis) and *K*_D_ values were determined using GraphPad Prism 10 and nonlinear fit of one-site specific binding.

### 2.8 Crystallization and structure determination

For crystallization of EBOV-GP trimers, the protein was concentrated to 8 mg/mL in a buffer containing 20 mM Tris (pH 8.0) and 150 mM NaCl. Crystals were grown at 20°C in a solution of 9% PEG 6000 and 100 mM sodium citrate (pH 5.2). Complex structures were obtained by soaking EBOV-GP crystals in a 5 mM solution of the target compound for a few minutes, followed by cryoprotection with 20% glycerol and rapid plunging into liquid nitrogen for storage before data collection. Diffraction data were collected at the Stanford Synchrotron Radiation Lightsource (SSRL) on beamline BL12-1. EBOV-GP crystals soaked with compound **4R** diffracted to a resolution of 2.59 Å. Data indexing, integration, and scaling were performed using HKL2000 (29). Structures were solved by molecular replacement (MR) with Phaser in Phenix (30) with PDB 6F6I as the MR model, and subsequent model building and refinement were conducted using Coot and Phenix.refine (31, 32). Structural quality was assessed with MolProbity (33), and further validation was performed using the PDB validation server. Data collection and refinement statistics are summarized in **Supplementary Table 1**.

## 3 Results

### 3.1 SuFExable tamoxifen analog design and validation of biological activity

**Figure 1:**
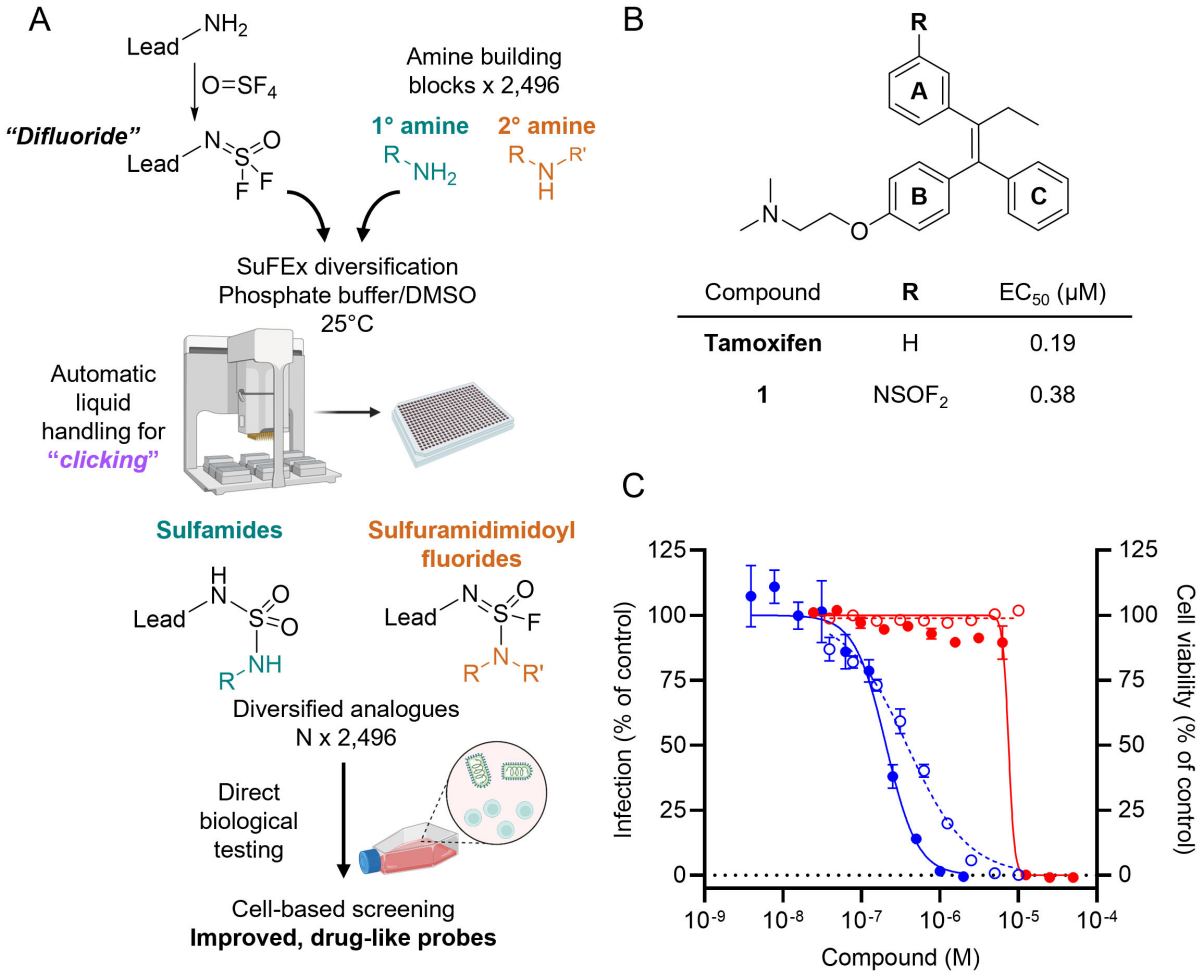
HTMC-based diversification of EBOV entry lead inhibitor tamoxifen. **(A)** Schematics of SuFEx-based HTMC workflow. **(B)** Chemical structure and EC_50_ of tamoxifen and the difluoride analog **1** against VSV-EBOV-GP (Vero cells). **(C)** Dose-response curves of VSV-EBOV-GP infection (blue) and Vero cell viability (red) for tamoxifen (●, solid lines) and compound **1** (○, dotted lines). Mean ± SD values are shown (n = 3).

The first step of the SuFEx-enabled HTMC workflow requires installation of a SuFExable difluoride moiety on the lead molecule (**Figure 1A**). This difluoride analog can be readily synthesized by reacting thionyl tetrafluoride (O=SF_4_) gas with the lead compound functionalized with a primary amine. A structural analysis of EBOV-GP protein + ligand complex was performed to determine the modification site on the lead molecule. Due to the absence of an experimentally determined crystal structure for the complex of EBOV-GP with tamoxifen, molecular docking was used to predict the binding mode of this ligand. For this purpose, we employed a crystal structure of toremifene (i.e. an analog of tamoxifen) bound to EBOV-GP (PDB entry: 5JQ7) as a template (34). Given the chemical structural similarity between tamoxifen and toremifene, we expected that both molecules would share similar binding conformations.

The docking suggests that tamoxifen aligns closely with the binding conformation of toremifene, preserving key interactions with crucial residues within the active site (**Supplementary Figure 1**). The aromatic rings B and C of tamoxifen adopt a similar orientation to the corresponding rings in toremifene, forming a π-stacking between these rings and Tyr517, as well as an ionic interaction between the protonated tertiary amine and the negatively charged side chain of Glu100. Additionally, the docking analysis highlights a solvent-exposed area at the *meta* position on the aromatic ring A, making it a prime candidate site for further functionalization. By targeting these solvent-exposed positions, we hypothesized that expanding the molecule into unoccupied regions of the binding pocket could form additional interactions with adjacent residues, thus enhancing both binding affinity and cellular anti-infective efficacy.

Guided by the predicted structure, we designed a synthetic route (**Supplementary Figure 2**) to introduce a difluoride group at the *meta* position of ring A of tamoxifen based on previous reports for the synthesis of analogs with the triphenylethylene core (25). The difluoride-containing analog **1** was successfully synthesized, and its chemical structure was verified by NMR and LC-MS analyses, as described in the **Supplementary Material**.

With analog **1** in hand, we assessed its antiviral activity and cytotoxicity, along with that of tamoxifen, using a virus-like particle (VLP) assay (26). The assay employs vesicular stomatitis virus (VSV) particles displaying EBOV-GP (VSV-EBOV-GP), in place of the native glycoprotein G, to infect Vero cells in culture. This approach obviated the need for high-security BSL4 facilities required for working with authentic filoviruses. The VSV-EBOV-GP also encodes an enhanced GFP, which allows for direct quantification of infected cells by fluorescence imaging. The use of a cell-based assay provides advantages for drug discovery, as it more accurately reflects the compounds’ activity in a biologically relevant environment and accelerates the profiling of cell-active compounds. This infection model enabled us to evaluate the inhibitor’s effectiveness in blocking the processing of viral GP, a crucial step for EBOV infection. Additionally, the VSV assay provided insight into whether the proposed structural modifications were tolerated by the lead compound without significantly compromising its activity.

The results showed that the lead compound tamoxifen has an EC_50_ value of 0.19 μM, while our difluoride-functionalized analog **1** exhibited an EC_50_ of 0.38 μM, with both values being within the same magnitude (**Figure 1B**). Importantly, compound **1** did not exhibit cytotoxicity up to 10 µM (**Figure 1C**). These findings indicate that the structural modifications to introduce the difluoride moiety do not critically reduce the biological activity of the lead compound, consistent with the docking predictions. The results support the effectiveness of the docking-guided design and validate the choice of the modification site on the tamoxifen aromatic ring.

### 3.2 SuFEx-enabled HTMC

**Figure 2:**
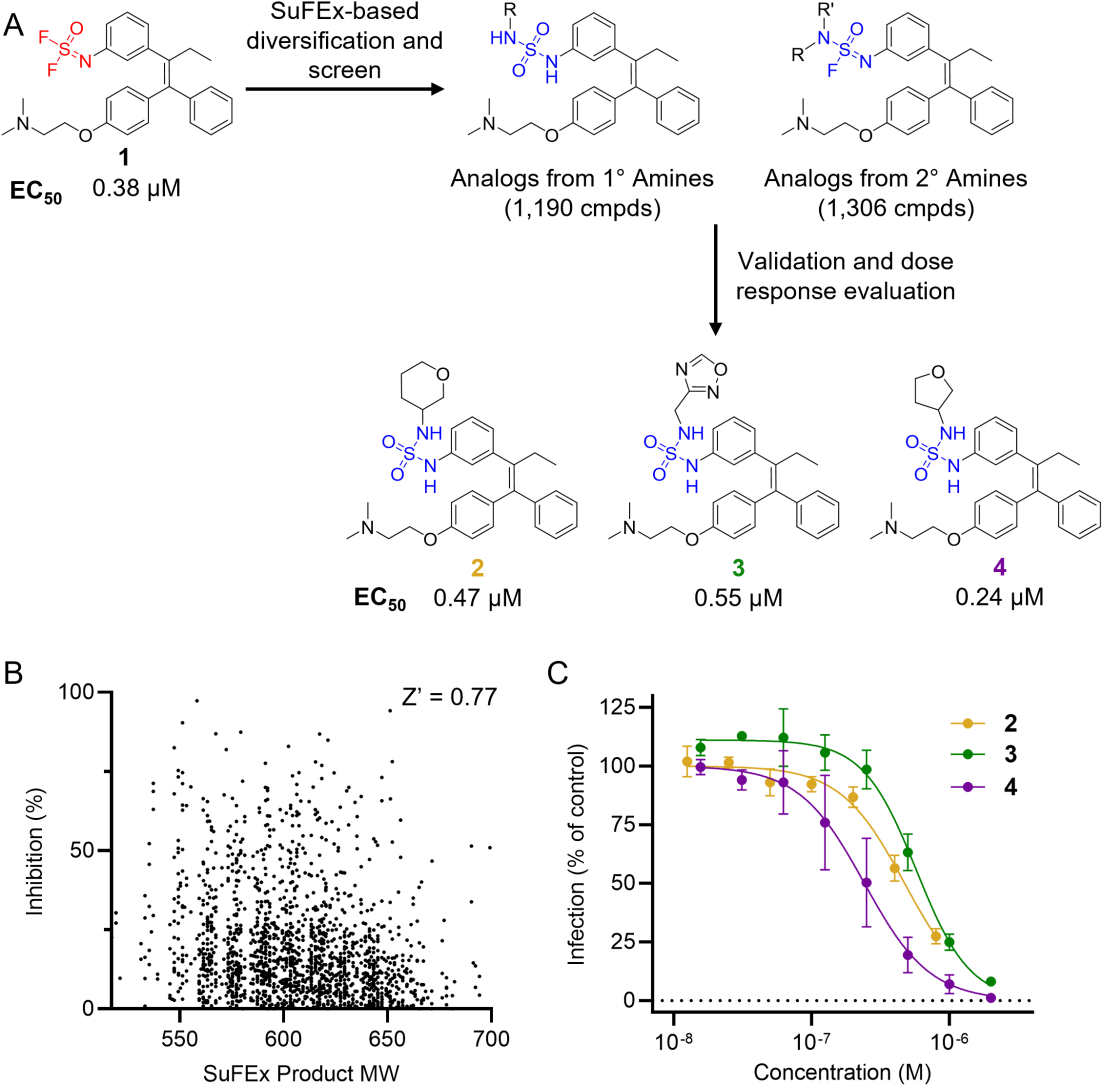
Reaction schematics and screening results of HTMC against EBOV entry. **(A)** HTMC workflow from a difluoride functionalized lead compound to the improved compounds. Chemical structures and EC_50_ (VSV-EBOV-GP, Vero cells) of representative hit molecules (compounds **2, 3**, and **4**) are shown. **(B)** Scatter plot for the tamoxifen-based HTMC library screening. Screening was performed at a small-molecule concentration of 200 nM. **(C)** Dose-response curves for representative hits identified from the HTMC library screening. Hit molecules were resynthesized in mg scale, purified, and chemically characterized, and their antiviral potency was measured against VSV-EBOV-GP using Vero cells. Mean ± SD values are shown (n = 3).

Once an appropriate SuFExable derivative of the lead compound has been identified, the next step in the HTMC workflow (**Figure 1A**) is the diversification reaction with an amine-fragment library. This strategy allows the preparation of a focused library of lead compound analogs with expanded diversity containing sulfamide or sulfuramidimidoyl fluoride linkages, from primary or secondary amines respectively (**Figure 2A**).

A library of 2,496 primary and secondary amine fragments were individually reacted with the difluoride-containing analog in 384-well plates overnight at room temperature using a 1:3 phosphate buffer:DMSO solvent mixture (see Materials and Methods for further details). The reaction conditions employed for diversification were determined in a preliminary assessment of the difluoride reactivity with a small set of representative amines. Reagents concentration, temperature, pH, and solvent composition were evaluated to identify the optimal conditions for generating a high-quality library, allowing us to screen the compound set without additional purification. The synthesis of the library was performed using an automated liquid handling robot Agilent Bravo BenchCel, enabling efficient generation of 2,496 analogs in a single batch. An LC-MS analysis of a randomly selected wells was performed to evaluate the quality of the library. The results indicated a generally high conversion rate, validating the suitability of the library for the D2B screen in the subsequent step.

The synthesized library was screened at 200 nM using the VSV-based EBOV entry assay. The library solutions were dispensed over Vero cells in 384-well plates. Cells treated with the compound were infected with VSV-EBOV-GP, and 16 hours post-infection, their nuclei were stained and then fixed. The infection rate was assessed by measuring eGFP expression levels, normalized to the number of nuclei. This approach allowed us to screen the compound set in singlicate with an assay Z′-factor of 0.77, indicating robust assay performance. The screening results are illustrated in **Figure 2B** as a scatter plot of % inhibition. After validating the hits by triplicate, the most potent molecules were manually synthesized in milligram quantities for further dose-dependent biochemical and cell-based characterization. This approach allowed us to confirm the efficacy and refine the profile of the most promising candidates. The structure of the representative hits synthesized for validation is shown in **Figure 2A** (compounds **2-4**), and the dose-response inhibition profile in **Figure 2C**. Compound 4 was identified as one of the top hits in the primary screening, with an EC_50_ value of 241 nM. Notably, compound **4** demonstrated an almost 2-fold increase in cellular antiviral potency compared to **1**.

### 3.3 Structure-activity relationships and biophysical analysis

To further understand the structure-activity relationships, we synthesized a small library of analogs of the identified molecules and subjected to dose-response antiviral and cytotoxicity studies. Specifically, we focused on modifications to the oxolane ring (**Table 1**, **Supplementary Figure 3**). These modifications included altering the ring size (**5**), investigating different substitution patterns on the oxolane ring (**6**, **7**), and replacing the oxolane with an N-methyl pyrrolidine ring (**8**) as well as other N-substituted analogs with varying polarity and chain lengths (**9 – 15**). Despite exploration of these broad structural modifications, no significant improvement in activity was observed with the ring changes. Since compound **4** was identified as a racemic mixture, we synthesized and evaluated both *R* and *S* enantiomers (**Table 1**). Interestingly, the *S*-configured compound (**4S**) exhibited a superior EC_50_ value of 92 nM, compared to its *R*-counterpart (**4R**) of 351 nM, indicating the importance of the chirality on the improved potency and specific interaction with GP-protein. The cytotoxicity studies showed variable CC_50_ values among the synthesized analogs. Compounds **4S** and **4R** did not show significant cytotoxicity below 10 µM with an excellent selectivity index (SI) of 150 for **4S** (**Table 1**).

**Table 1:** Structure-activity relationships of compound 4 analogs against VSV-EBOV-GP.

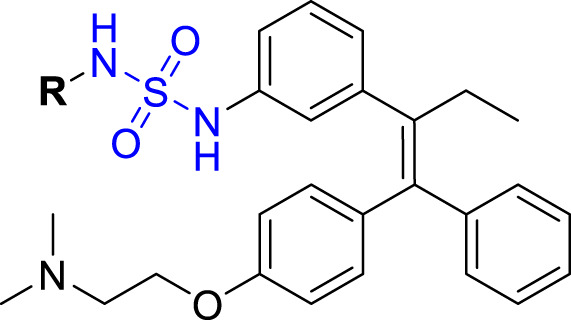										
Cmpd	R	EC_50_ ^a^ (nM)	CC_50_ ^b^ (µM)	SI^c^		Cmpd	R	EC_50_ ^a^ (nM)	CC_50_ ^b^ (µM)	SI^c^
**4**	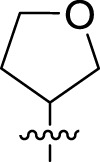	240	n.d.	n.d.		**9**	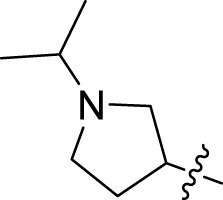	790	3.4	4.3
**4R**	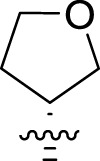	351	15	43		**10**	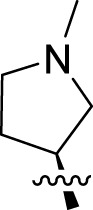	664	3.0	4.5
**4S**	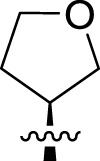	92	14	150		**11**	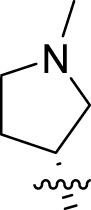	575	3.6	6.3
**5**	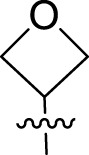	156	6.8	44		**12**	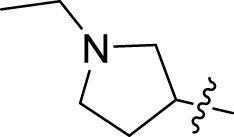	841	4.0	4.8
**6**	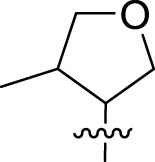	201	14	70		**13**	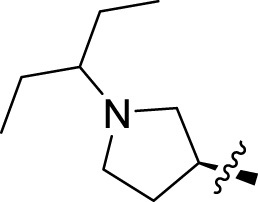	813	5.3	6.5
**7**	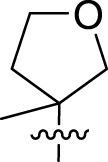	268	8.4	31		**14**	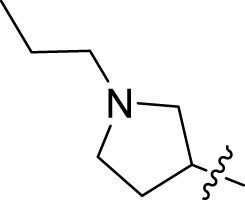	618	3.1	5.0
**8**	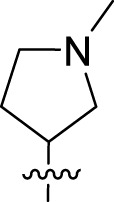	145	2.0	14		**15**	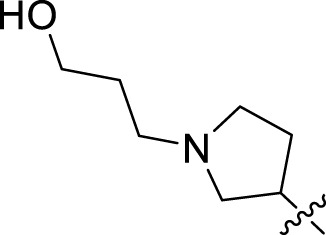	1720	7.7	4.5

a EC_50_ values were measured against VSV-EBOV-GP (Vero cells).

b CC_50_ values were measured against Vero cells using Promega^®^ CellTiter-Glo^®^ 2.0. Reported EC_50_ and CC_50_ values are the average of triplicate measurements with at least two data points above and at least two below the EC_50_.

c SI: Selectivity index = CC_50_/EC_50_. n.d. = not determined.

**Figure 3:**
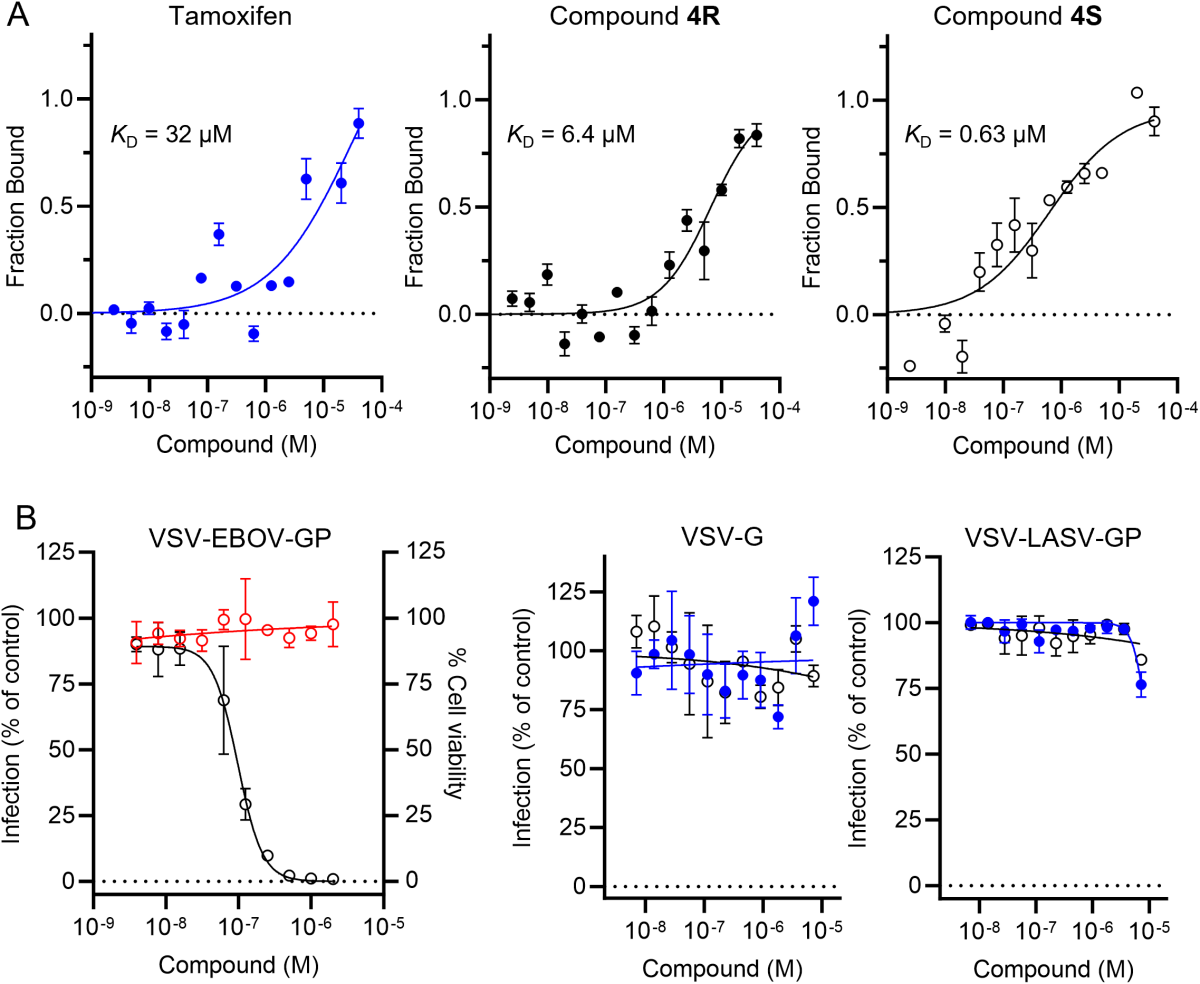
Characterization of the top tamoxifen analog **4S** identified from the HTMC campaign. **(A)** The biophysical affinity of tamoxifen, **4R** and **4S** towards EBOV-GP as measured by MST. Error bars indicate the standard deviation of three technical replicates (n = 3). **(B)** Dose-response curves of VSV-EBOV-GP infection (○) and Vero cell viability (○) for compound **4S** (left panel). Tamoxifen (●) and compound **4S** (○) were tested for their ability to inhibit VLPs displaying the VSV-G glycoprotein (middle panel) and LASV-GP (right panel). Mean ± SD values are shown (n = 3).

To confirm the direct interaction of **4R** and **4S** with EBOV-GP, the equilibrium dissociation constants (*K*_D_) for the interaction inhibitor-protein were measured. The recombinant protein was expressed in Expi293S cells and purified using Ni-NTA affinity and size-exclusion chromatography. Then, we evaluated the interaction of both enantiomers as well as the parent tamoxifen with EBOV-GP by microscale thermophoresis (MST). As shown in **Figure 3A**, **4S** displayed a stronger binding affinity against EBOV-GP, with a *K*_D_ value of 0.63 µM, compared to **4R** with a *K*_D_ value of 6.4 µM. In comparison, the corresponding value for tamoxifen was 32 µM. This trend mirrors the observed differences in cellular potency, where **4S** demonstrated enhanced efficacy at low submicromolar concentrations in blocking EBOV entry, while **4R** required higher concentrations to achieve a comparable effect (**Table 1**). It is worth noting the 50-fold affinity enhancement in the biochemical assay between **4S** and tamoxifen, providing further validation of improved potency of the inhibitor developed through our HTMC platform.

**Figure 4:**
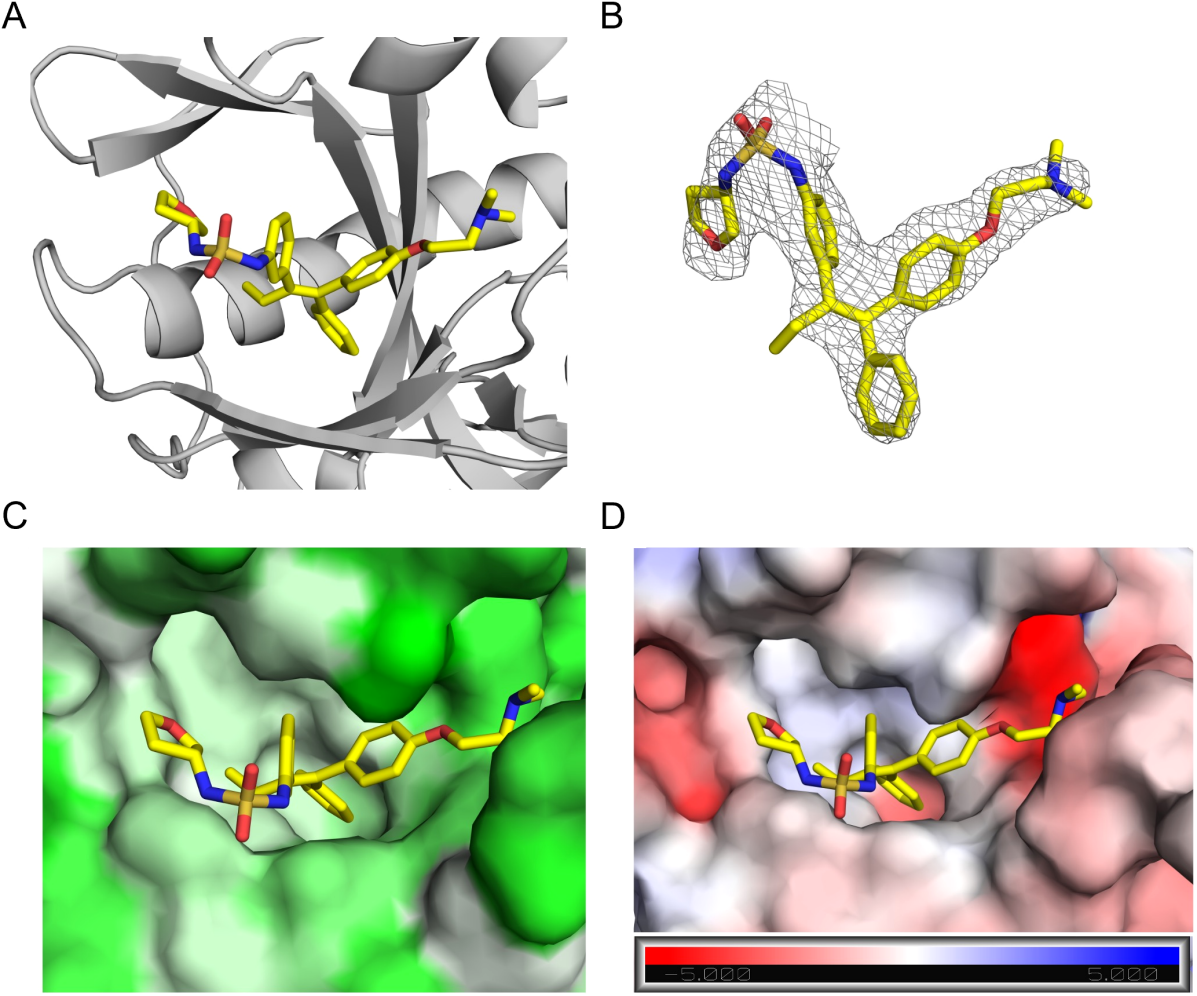
Structure of EBOV-GP in complex with compound **4R**. **(A)** Crystal structure of EBOV-GP trimer bound to **4R**. The compound is depicted as sticks, and the protein as a cartoon representation. **(B)** 2Fo-Fc electron density map contoured at 1 sigma for **4R**. **(C) 4R** bound within the hydrophobic binding pocket of EBOV-GP. The surface is colored according to hydrophobicity, with pale green indicating the most hydrophobic regions and green the least hydrophobic. **(D)** Electrostatic surface potential of the binding pocket, colored from red (most negative) to blue (most positive), with **4R** bound. The color scale for electrostatics is shown at the bottom of the figure.

We then determined the X-ray structure of **4R** in complex with EBOV-GP to 2.59 Å resolution (**Figure 4**, **Supplementary Table 1**). The electron density for **4R** is well defined (**Figure 4B**) and the compound binds within the extensive hydrophobic cavity of EBOV-GP, a known target site for other inhibitors. It adopts a similar pose to its analog, toremifene, with conserved interactions involving the three aromatic rings and the dimethylethanamine group of the parent scaffold (34). Compared to toremifene, **4R** occupies a larger portion of the available cavity, with its additional moiety engaging a hydrophobic subpocket, potentially contributing to its improved binding. Unfortunately, we were unable to obtain a structure for **4S**, as it renders the crystal unstable, possibly due to conformational changes when the compound is soaked into the crystal, and leads to poor diffraction. Nevertheless, the complex structure of EBOV-GP and **4R** validates the docking model of our lead molecule and provides structural insights into the improved potency of the developed molecules.

Finally, we evaluated the viral selectivity and specificity of compound **4S** by comparing the inhibitory activity against VSV-EBOV-GP to VSV-G and VSV particles displaying LASV-GP (VSV-LASV-GP, **Figure 3B**). Tamoxifen and our compound **4S** showed selective inhibition of VSV-EBOV-GP but not VSV-G or VSV-LASV-GP, indicating that its antiviral activity is specific to EBOV-GP. This result supports the hypothesis that compound **4S** does not induce non-specific effects on viral entry mechanisms shared by multiple viruses or inhibit proteins involved in later stages of the EBOV infection, such as cathepsins, which are known to mediate viral entry through endosomal processing. Furthermore, the lack of activity in VSV-G and VSV-LASV-GP models reduces the likelihood that the compound’s mechanism involves general endosomal disruption or interference with acidic environments. This selectivity, in concert with the chiral preference of **4S** over its *R*-isomer in binding EBOV-GP, suggests that the compound engages specific molecular interactions with EBOV-GP that are crucial for its activity.

## 4 Discussion

In this study, we leveraged our unique SuFEx-based HTMC platform to generate a large-scale library of tamoxifen analogs with the aim of identifying potent inhibitors of EBOV entry. This approach enabled a rapid SAR analysis that accelerated the identification of novel EBOV inhibitors, highlighting the potential of HTMC workflow as a powerful tool for antiviral drug discovery. Our inhibitor discovery efforts were motivated by the need for effective small-molecule capable of modulating EBOV infection, especially given that current treatment options primarily include vaccines and monoclonal antibodies. While these approaches have shown promise, there remains an unmet need for potent small molecules that can be translated to clinical application. This work also demonstrated a successful application of our SuFEx-based HTMC platform for accelerated optimization of a potential antiviral compound, as we have shown previously for other biological targets (17–21).

To develop this strategy, we employed tamoxifen as a lead scaffold to design an analog with a required SuFExable hub for accelerated diversification. Although other analogs of tamoxifen, such as toremifene and clomiphene, have been extensively characterized as EBOV entry inhibitors (24), tamoxifen was the most suitable scaffold in terms of synthetic design to access to the required functionalized difluoride. The SuFEx-enabled HTMC approach applied here is especially well-suited to drug discovery for infectious diseases such as EVD, where the iterative cycle of lead optimization can be a significant bottleneck. Previous efforts on the optimization of tamoxifen analogs of these compounds have relied on traditional medicinal chemistry. Our SuFEx-based D2B platform eliminates the need for extensive purification. The biocompatible conditions employed for diversification enabled crude products to be tested directly in a cell-based assay. Furthermore, the method facilitated exploration of structural modifications that improve binding affinity and potency, as evidenced by the development of compounds with targeted changes that demonstrated improved antiviral efficacy without compromising cellular viability.

The design basis of our tamoxifen analog functionalized with a difluoride (compound **1**) stems from the binding interactions observed in the predicted complex of tamoxifen with EBOV- GP, which suggested sites for functionalization to enhance binding affinity. By targeting the unoccupied pocket, we aimed to improve interaction around the binding pocket. The crystal structure of EBOV-GP with **4R** provided further validation to our docking approach for lead design.

This approach led to compound **4S** that demonstrated sub-micromolar inhibition of EBOV entry and a 50-fold improvement in binding affinity against EBOV-gp over tamoxifen as measured by MST. The stronger binding of **4S** to EBOV-GP, as reflected in its enhanced *K*_D_, is consistent with its superior cellular potency, suggesting that improved target affinity is likely to contribute to its increased functional activity. However, it is worth noting that the improvement of cellular potency is around 2-fold compared with tamoxifen. Elucidating the apparent discrepancy between the improvement in biophysical affinity and cellular potency is the subject of further study.

Our structure-activity relationship put in evidence the chiral selectivity of the hit identified, with the analog **4R** showing less potency than its corresponding *S* enantio-counterpart, as measured by biophysical affinity as well as cellular antiviral activity. The significance of stereochemistry in **4S** exemplifies the precision achievable with this SAR-guided platform and highlights the potential for designing small molecules for enhanced interactions with viral proteins. These findings underscore the importance of chiral optimization in the development of effective EBOV entry inhibitors and validate **4S** as a particularly promising lead compound.

Unfortunately, we could not determine the crystal structure of the most active compound, **4S**. However, an analysis of the pose adopted by compound **4R** in the binding pocket of EBOV-GP revealed that the newly introduced oxolanyl ring, resulting from the SuFEx reaction, is positioned near the region where the β13-β14 loop (residues 190 to 214) is expected. This region has also been observed to be disordered in previous structures (34, 35). Structural analyses of toremifene bound to EBOV-GP have shown that this scaffold binds at the same site at the entrance of the binding pocket by expelling the DFF lid (residues 192–195) and positioning the A ring of the scaffold (**Figure 1B**) within this region (34). We propose that this flexible fragment could be modulating the enantioselectivity of the inhibitors identified in this work. However, additional experiments are required to verify this hypothesis.

In conclusion, this study demonstrates the effectiveness of our SuFEx-based HTMC platform in identifying promising EBOV entry inhibitors, contributing to the growing therapeutic modalities available for tackling the challenges associated with viral infections. This work paves the way for future studies to further optimize these lead compounds and assess their *in vivo* efficacy in EBOV infection. More broadly, the results of our HTMC workflow underscore the capability of this technology to accelerate the design and development of drug candidates in the field of viral infection, immunology, and autoimmune diseases.

## Data Availability

The original contributions presented in the study are included in the article/**Supplementary Material**. The data presented in the study is deposited in the RCSB PDB repository, accession number 9NNU. Further inquiries can be directed to the corresponding author.
