# Supplementary material for: SuFEx-enabled high-throughput medicinal chemistry for developing potent tamoxifen analogs as Ebola virus entry inhibitors
Source: Front Immunol. 2025 Apr 28;16:1533037. doi: 10.3389/fimmu.2025.1533037 (PMC12066687; doi:10.3389/fimmu.2025.1533037)
Supplement: Supplementary file 1 [file DataSheet1.pdf]

Supplementary Material

Table of Contents

Supplementary Figure 1 2
Supplementary Figure 2 3
Supplementary Figure 3 4
Supplementary Table 1 5
Chemistry 6
NMR data: ^{1}H, ^{13}C, ^{19}F 9
References 14

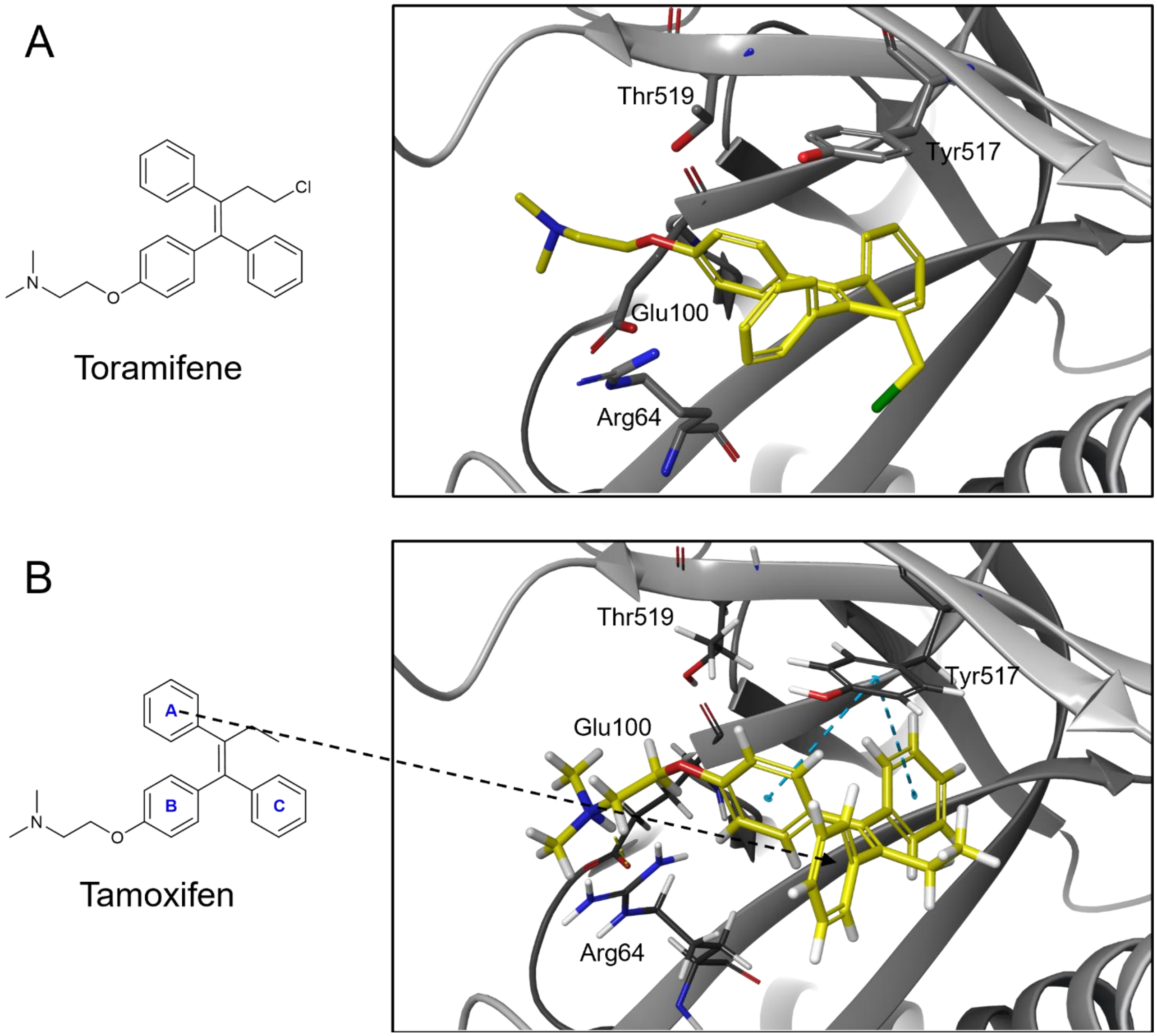

Supplementary Figure 1. Comparative poses between toremifene and tamoxifen with EBOV-GP. The ligand is depicted as sticks, and the protein as a cartoon representation. Relevant residues of the protein are shown as sticks. **(A)** Crystal structure of toremifene bound to EBOV-GP (from PDB 5JQ7). **(B)** Docking pose of tamoxifen in the binding site of EBOV-GP. Hydrogen bond and pi-pi stacking interactions between EBOV-GP and the docked compound are in yellow and light-blue, respectively. The black arrow indicates the aromatic ring **A** with solvent-exposed positions.

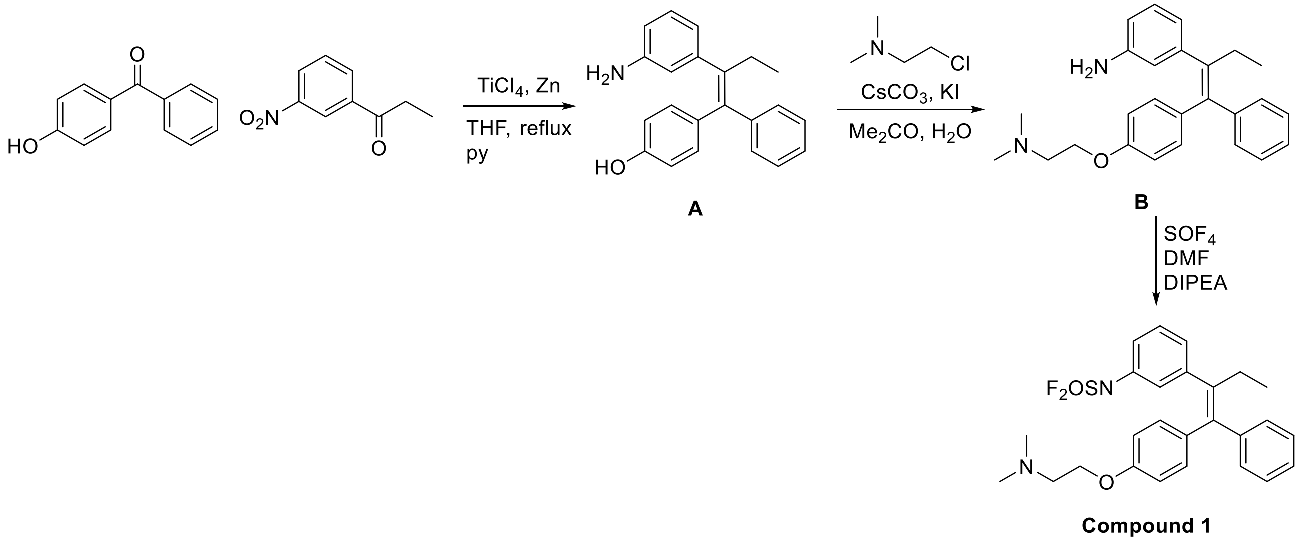

Supplementary Figure 2. Synthetic route for difluoride **1**.

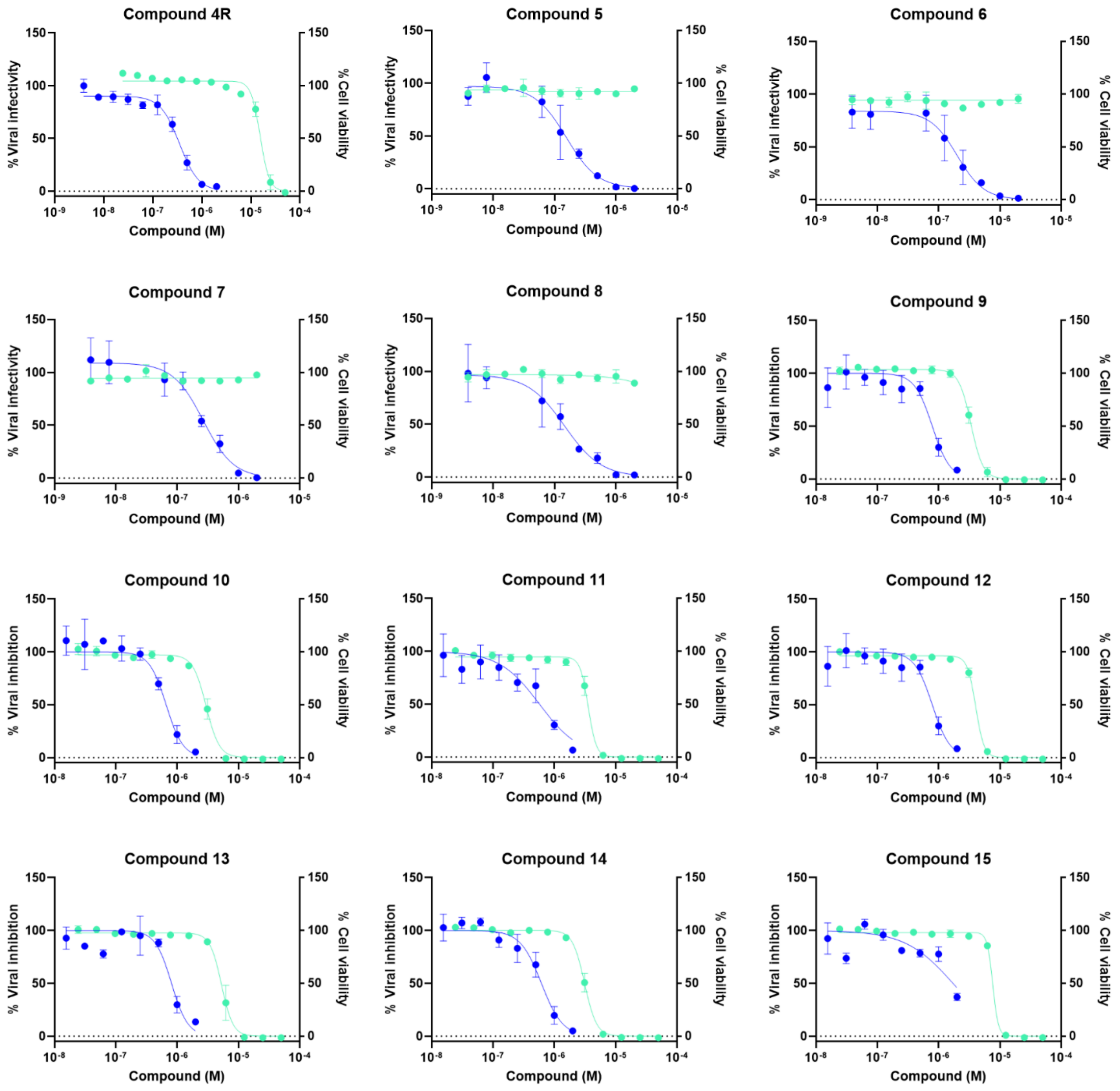

Supplementary Figure 3. Dose-response curves of VSV-EBOV-GP infection (blue) and Vero cell viability (green) for compounds **4 - 15**. Mean ± SD values are shown (n = 3).

Supplementary Table 1. X-ray data collection and refinement statistics.

**Data Collection**	**EBOV-GP-4R**
Beamline	SSRL BL12-1
Wavelength (Å)	0.979
Resolution (Å)	30.00 - 2.59<br>(2.64 – 2.59)
Space group	R32:h
Unit cell a, b, c (Å)	114.2 114.2 306.4
α, β, γ (°)	90 90 120
Total reflections	224,540
Unique reflections	24,322
Multiplicity	9.2 (9.0)
Completeness (%)	99.5 (99.9)
Mean I/sigma(I)	22.4 (0.9)
Rsym	0.21 (>3)
Rpim	0.07 (1.4)
CC1/2	0.99 (0.45)
**Refinement Statistics**	
Resolution (Å)	29.27 - 2.59
Reflections total / Rfree	24,246 / 2014
R_{cryst} / R_{free}	0.22 / 0.24
No. of copies in ASU	1
Number of atoms	3007
macromolecules	2863
ligands	144
solvent	-
Average B-factor ($Å^2$)	76
macromolecules	75
ligands	95
Wilson B-factor ($Å^2$)	68
**RMSD from ideal geometry**	
Bond angle (°)	0.61
Bond length (Å)	0.003
**Ramachandran statistics (%)**	
Favored	98.29
Allowed	1.71
**PDB Code**	**9NNU**

Statistics for the highest-resolution shell are shown in parentheses.

Chemistry

All reagents and solvents were purchased from commercial suppliers and were used without further purification. 1-(3-nitrophenyl)propan-1-one was purchased from AmBeed and (4-hydroxyphenyl)(phenyl)methanone from 1Pluschem. ^{1}H, ^{13}C and ^{19}F NMR spectra were collected at 298K using a Bruker 600 spectrometer with chemical shifts reported relative to residual deuterated solvent peaks or tetramethylsilane internal standard. $CFCl_3$ was used as an internal standard for ^{19}F-NMR. Reactions were monitored on TLC plates (silica gel 60, F254 coating, EMD Millipore, 1057150001), and spots were monitored under UV light (254 mm) and/or ninhydrin staining. The purity of the compounds that were tested in the assay was >95% based on ^{1}H-NMR and reverse-phase high-performance liquid chromatography (HPLC)-UV (Agilent LC-MSD system with 1290MCT column oven, 1290 Multisampler, 1290 High Speed Pump, 1290 DAD FS, LC/MSD, reverse phase HPLC column Agilent InfinityLab Poroshell 120 EC-C18 (2.1 x 50 mm, particle size 1.9 μm)) on monitoring absorption at 254 nm. The HPLC gradient method consisted of an aqueous phase (Milli-Q water with 0.1% formic acid (FA)) and an organic phase (acetonitrile with 0.1% FA) with a 0.50 mL/min flow. The first step consisted of 95% aqueous and 5% organic phases followed by a 4.0 min gradient to 100% organic phase. A subsequent 30-second step of 100% organic phase was followed by a 30-second gradient to 95% aqueous and 5% organic phase. Preparative HPLC was performed with Waters2545 Binary Gradient Module, Waters515 HPLC Pump, Waters SFO System Fluidics organizer, Waters2489 UV/Visible Detector, Waters2424 ELS Detector, Waters QDa Detector, and Waters3767 Sample Manager using a C18 reverse phase column (Waters, Waters X Bridge BEH C18 OBD Prep Column, 19 x 250 mm, 5 μm).

(*Z*)-4-(2-(3-aminophenyl)-1-phenylbut-1-en-1-yl)phenol (**A**)

A well-dried 3-neck round bottom flask was loaded with powder zinc (2.380 g, 36.4 mmol, 10 eq) and dry THF (50 mL). The flask was purged with argon and cooled in an ice bath. $TiCl_4$ (2.2 mL, 20 mmol, 5.5 eq) was added slowly and stirred for 5 min. The system was heated to reflux at 69 °C for 3 h. The mixture was cooled in an ice bath, and pyridine (0.74 mL, 9.1 mmol, 2.5 eq) was added dropwise. Then, a solution of 1-(3-nitrophenyl)propan-1-one (657 mg, 3.67 mmol, 1.0 eq) and (4-hydroxyphenyl)(phenyl)methanone (870 mg, 4.39 mmol, 1.2 eq) in dry THF (15 mL) was added to the system. The reaction mixture was covered to darkness with aluminum foil and refluxed at 69 °C overnight. The next day, TLC analysis (hexane/ethyl acetate 6:4) showed complete conversion of the starting materials and a main new spot with R_f 0.58 stained positively with ninhydrin. The reaction mixture was diluted with dichloromethane (50 mL), filtered through a celite bed, and washed with K_2CO_3 (10%, 100 mL) and brine (100 mL). The organic phase was dried with $MgSO_4$, filtered, and concentrated to dryness. The residue was further purified by flash chromatography (hexane: ethyl acetate gradient) to afford compound **A** (810 mg, 70%). ^{1}H-NMR (600 MHz, MeOH-d_4) 7.23 (t, J = 7.4 Hz, 2H), 7.14 (t, J = 7.2 Hz, 1H), 7.08 (d, J =7.8 Hz, 2H), 6.84 (t, J = 7.8 Hz, 1H), 6.60 (d, J = 8.5 Hz, 2H), 6.50 (brs, 1H), 6.44 (dd, J = 7.9, 1.6 Hz, 1H), 6.42 (d, J = 7.5 Hz, 1H), 6.31 (d, J = 8.9 Hz, 2H), 2.27 (q, J = 7.4 Hz, 2H), 0.80 (t, J = 7.4 Hz, 3H). ^{13}C-NMR (150 MHz, MeOH-d_4) 156.4, 147.4, 145.4, 144.9, 142.3, 139.5, 135.7, 132.8, 135.5, 130.5, 129.6, 129.1, 127.5, 121.9, 118.5, 115.1, 114.8, 30.0, 13.9. MS (ESI, positive): calcd. for $[C_{22}H_{22}NO]^+$ 316.2, found 316.3.

(Z)-3-(1-(4-(2-(dimethylamino)ethoxy)phenyl)-1-phenylbut-1-en-2-yl)aniline (**B**)

Compound **A** (108 mg, 0.34 mmol) was dissolved in dry THF (4 mL), and 2-chloro-N,N-dimethylethan-1-amine hydrochloride (79 mg, 0.55 mmol, 1.60 eq), potassium iodide (8.9 mg, 0.05 mmol, 0.16 eq) and cesium carbonate (346 mg, 1.06 mmol, 3.10 eq) were added. The reaction was stirred at 80 °C overnight. The next day, TLC analysis (hexane/ethyl acetate 6:4) showed complete conversion of the starting material. The reaction mixture was diluted in dichloromethane (10 mL) and washed with water (10 mL x 2) and brine (10 mL). The organic phase was dried with $MgSO_4$, filtered, and concentrated to dryness. The reaction mixture was purified via preparative HPLC using a H_2O-MeCN gradient. A fraction containing the target molecule was lyophilized to afford compound **B** (61 mg, 46%). ^{1}H-NMR (600 MHz, DMSO-d_6) 7.37 (t, J = 7.7 Hz, 2H), 7.28 (t, J = 7.3 Hz, 1H), 7.23 (dd, J =8.3 Hz, 2H), 7.00 (m, 1H), 6.87 (d, J = 8.7 Hz, 2H), 6.64 (d, J = 8.8 Hz, 2H), 6.63 (brs, 1H), 6.59 (m, 2H), 4.18 (t, J = 4.2 Hz, 2H), 3.38 (t, J = 5.0 Hz, 2H), 2.82 (s, 6H), 2.34 (q, J = 7.4 Hz, 2H), 0.88 (t, J = 7.4 Hz, 3H). ^{13}C-NMR (150 MHz, DMSO-d$_6$) 156.7, 144.6, 144.4, 142.8, 138.9, 137.7, 132.5, 130.1, 129.7, 129.3, 127.7, 122.6, 119.0, 115.7, 114.5, 62.7, 57.3, 44.1, 29.7, 13.8. MS (ESI, positive): calcd. for $[C_{26}H_{31}N_2O]^+$ 387.2, found 387.4.

(Z)-(3-(1-(4-(2-(dimethylamino)ethoxy)phenyl)-1-phenylbut-1-en-2-yl)phenyl)sulfurimidoyl difluoride (compound **1**)

The method for preparing the iminosulfur oxydifluoride **1** is adapted from Li et al. (1). In a 25 mL round bottom flask, compound **B** (103 mg, 0.27 mmol) and DIPEA (185 µL, 1.06 mmol, 4.0 equiv) were dissolved in anhydrous acetonitrile (5.3 mL). Sealed with a rubber septum, the flask was evacuated and backfilled with thionyl tetrafluoride gas (~25 mL). The reaction was monitored by LC-MS analysis, which showed complete conversion of the starting material after 30 minutes. The volatiles were removed, and the crude was purified by preparative HPLC chromatography to give the target iminosulfur oxydifluoride **1** as a clear syrup (92.3 mg, 74%). ^{1}H-NMR (600 MHz, DMSO-d_6) 7.32 (t, J = 7.7 Hz, 2H), 7.24 (t, J = 7.9 Hz, 2H), 7.15 (d, J =8.1 Hz, 2H), 7.08 (brd, J =7.8 Hz, 1H), 6.94 (dd, J = 8.0, 0.9 Hz, 1H), 6.83 (brs, 1H), 6.69 (d, J = 8.3 Hz, 2H), 6.59 (d, J = 8.8 Hz, 2H), 3.92 (t, J = 5.4 Hz, 2H), 3.25 (m, 2H), 2.31 (s, 6H), 2.31 (m, 2H), 0.80 (t, J = 7.5 Hz, 3H). ^{13}C-NMR (150 MHz, DMSO-d_6) 156.9, 144.2, 143.3, 140.1, 139.4, 135.3, 131.7, 130.1, 129.3, 128.8, 127.8, 127.3, 125.3, 121.9, 114.1, 64.8, 57.3, 45.0, 28.6, 13.7. ^{19}F-NMR (471 MHz, DMSO-d_6) 46.85. MS (ESI, positive): calcd. for $[C_{26}H_{29}F_2N_2O_2S]^+$ 471.2, found 471.4.

Representative procedure for iminosulfur oxydifluoride and amine reactions

To a solution of compound **1** (4.0 mg, 8.5 µmol) in acetonitrile (85 µL) were added the amine hydrochloride (47 µmol, 5.5 eq) in PBS (85 µL) and DIPEA (55 µmol, 6.5 eq). The reaction was stirred overnight at room temperature in darkness. This solution was filtered through 0.22 µm filter and purified on preparative HPLC to give the pure target compound.

N-{3-[(1Z)-1-{4-[2-(dimethylamino)ethoxy]phenyl}-1-phenylbut-1-en-2-yl]phenyl}{[(3S)-oxolan-3-yl]amino}sulfonamide (compound **4S**)

Yield = 60%. ^{1}H NMR (600 MHz, MeOH-d_4) δ 7.25 (t, J = 7.6 Hz, 2H), 7.17 (t, J = 7.7 Hz, 1H), 7.10 (d, J = 7.5 Hz, 2H), 6.99 (t, J =7.6 Hz, 1H), 6.88 (brs, 1H), 6.86 (d, J = 8.1 Hz, 1H), 6.77 (d, J = 8.4 Hz, 2H), 6.70 (d, J = 7.5 Hz, 1H), 6.59 (d, J = 8.4 Hz, 2H), 4.12 (m, 2H), 3.75 (m, 1H), 3.68 (m, 1H), 3.68 (m, 1H), 3.60 (m, 1H), 3.41 (m, 2H), 3.29 (dd, J = 8.8, 4.5 Hz, 1H), 2.83 (s, 6H), 2.34 (m, 2H),

1.96 (m, 1H), 1.63 (m, 1H), 0.82 (t, *J* = 7.4 Hz, 3H). ^{13}C NMR (150 MHz, MeOH-d_4) δ 155.7, 143.5, 143.3, 141.4, 138.4 x 2, 136.7, 131.7, 128.9, 128.3, 127.9, 126.4, 124.3, 119.1, 115.7, 113.3, 72.3, 66.4, 61.4, 56.3, 53.2, 42.4, 32.3, 28.5, 12.4. MS (ESI, positive): calcd. For $[C_{30}H_{38}N_3O_4S]^+$ 536.3, found 536.5. Purity (HPLC-UV 254 nm): >98%.

N-{3-[(1*Z*)-1-{4-[2-(dimethylamino)ethoxy]phenyl}-1-phenylbut-1-en-2-yl]phenyl}{[(3*R*)-oxolan-3-yl]amino}sulfonamide (compound **4R**)

Yield = 60%. ^{1}H NMR (600 MHz, MeOH-d_4) δ 7.25 (t, *J* = 7.6 Hz, 2H), 7.17 (t, *J* = 7.7 Hz, 1H), 7.10 (d, *J* = 7.5 Hz, 2H), 6.99 (t, *J* =7.6 Hz, 1H), 6.88 (brs, 1H), 6.86 (d, *J* = 8.1 Hz, 1H), 6.77 (d, *J* = 8.4 Hz, 2H), 6.70 (d, *J* = 7.5 Hz, 1H), 6.59 (d, *J* = 8.4 Hz, 2H), 4.12 (m, 2H), 3.75 (m, 1H), 3.68 (m, 1H), 3.68 (m, 1H), 3.60 (m, 1H), 3.41 (m, 2H), 3.29 (dd, *J* = 8.8, 4.5 Hz, 1H), 2.83 (s, 6H), 2.34 (m, 2H), 1.96 (m, 1H), 1.63 (m, 1H), 0.82 (t, *J* = 7.4 Hz, 3H). ^{13}C NMR (150 MHz, MeOH-d_4) δ 155.7, 143.5, 143.3, 141.4, 138.4 x 2, 136.7, 131.7, 128.9, 128.3, 127.9, 126.4, 124.3, 119.1, 115.7, 113.3, 72.3, 66.4, 61.4, 56.3, 53.2, 42.4, 32.3, 28.5, 12.4. MS (ESI, positive): calcd. For $[C_{30}H_{38}N_3O_4S]^+$ 536.3, found 536.4. Purity (HPLC-UV 254 nm): >98%.

NMR data: ^{1}H, ^{13}C, ^{19}F

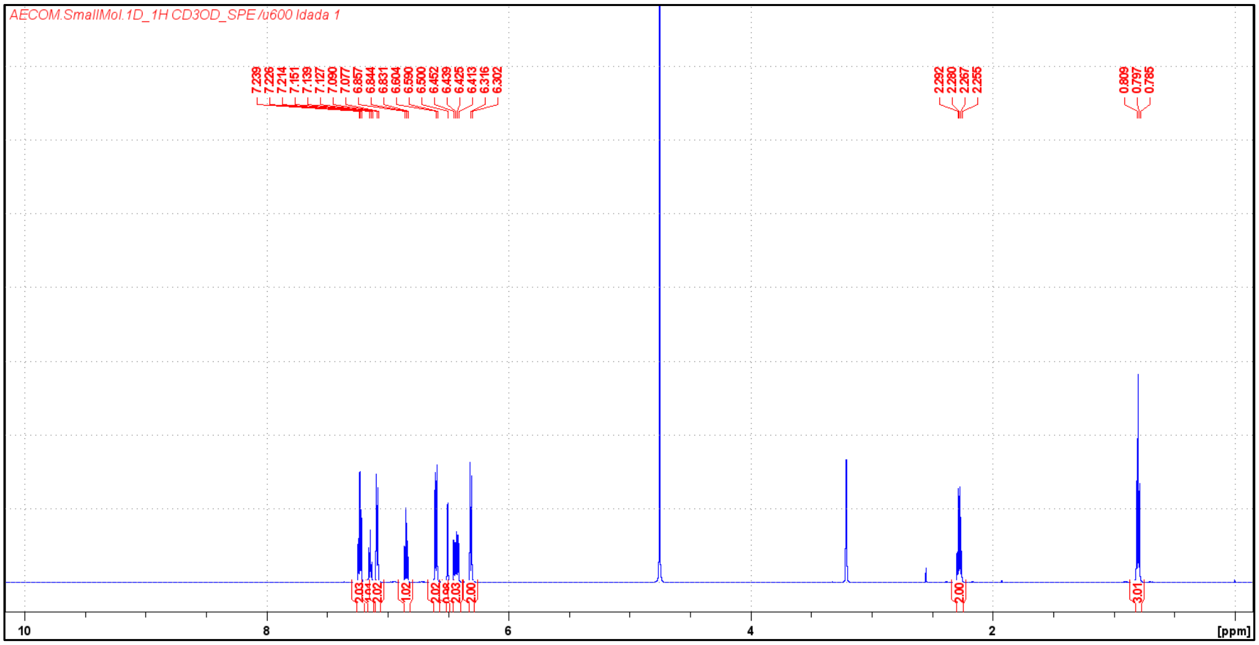

^{1}H-NMR (600 MHz, MeOH-d_4) of compound **A**.

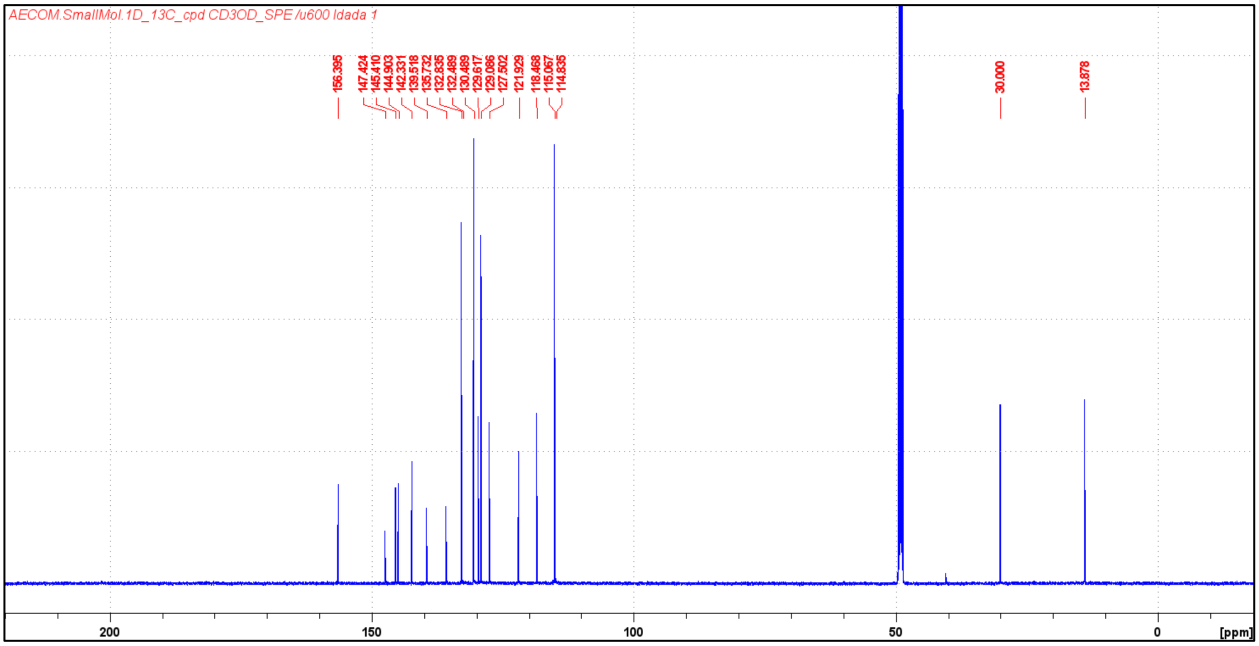

^{13}C-NMR (150 MHz, MeOH-d_4) of compound **A**.

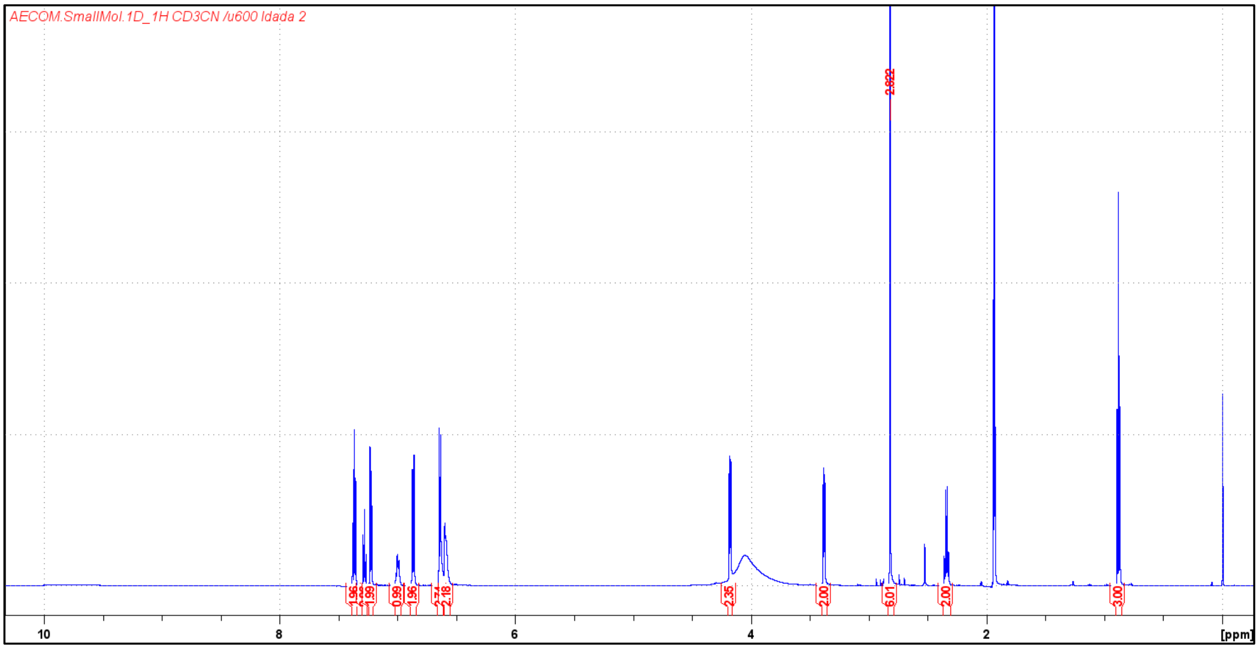

^{1}H-NMR (600 MHz, DMSO-d_6) of compound **B**.

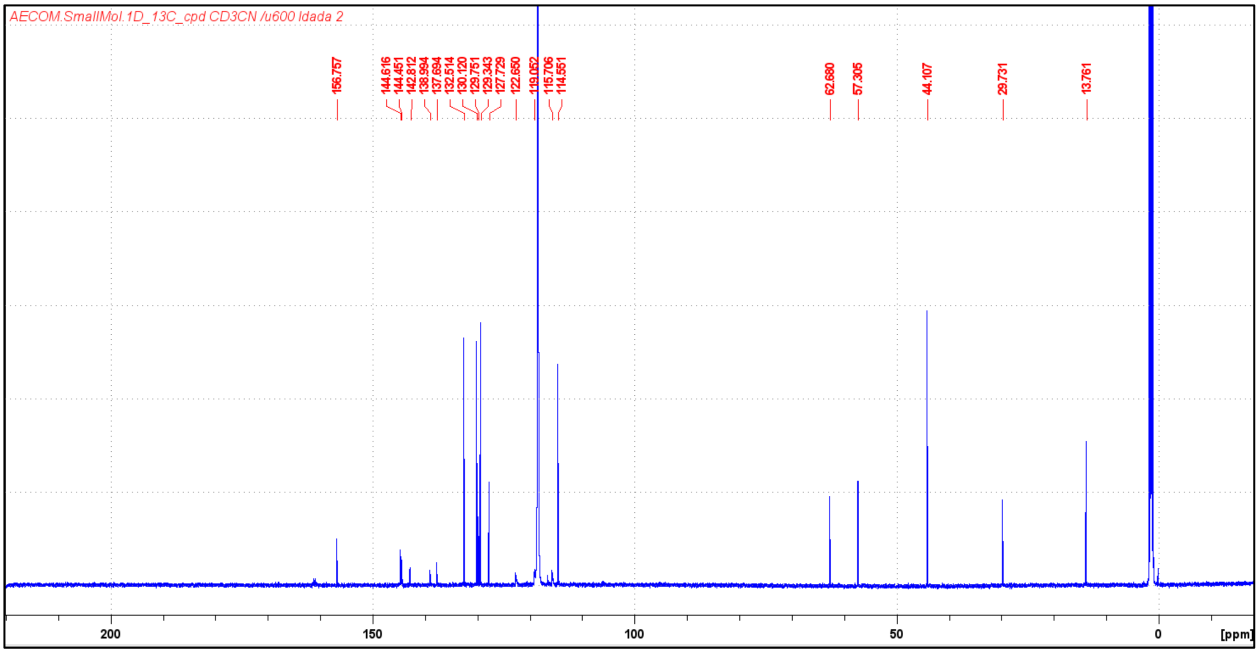

^{13}C-NMR (150 MHz, DMSO-d_6) of compound **B**.

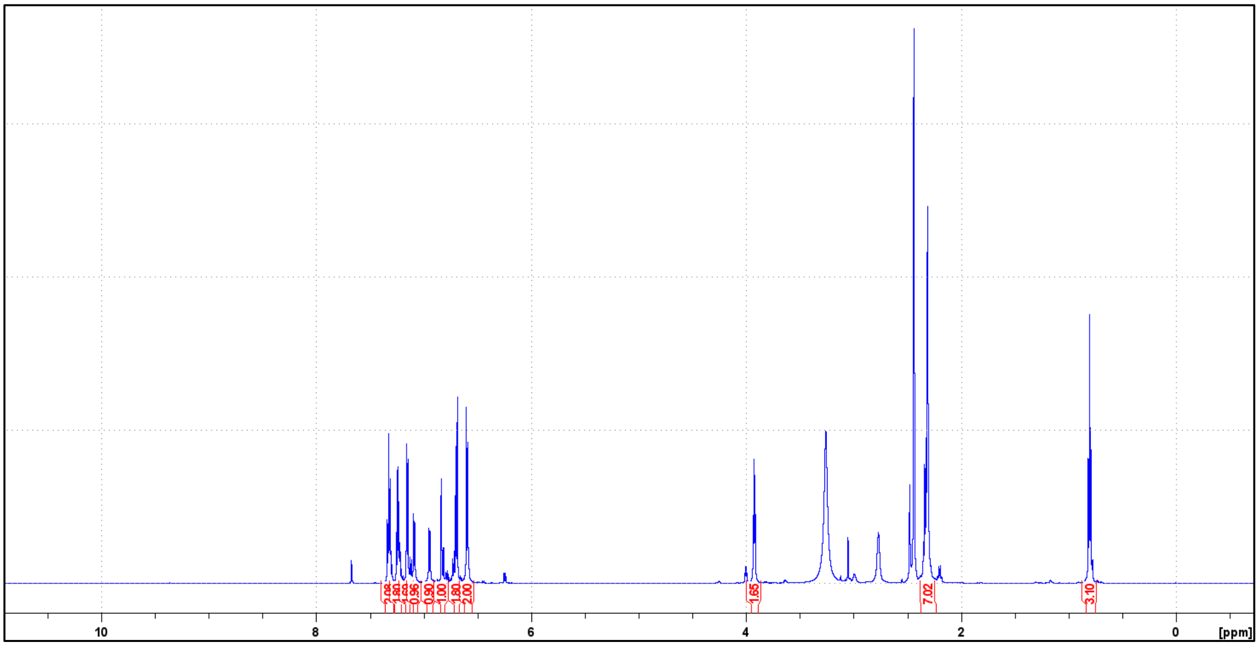

^{1}H-NMR (600 MHz, DMSO-d_6) of compound **1**.

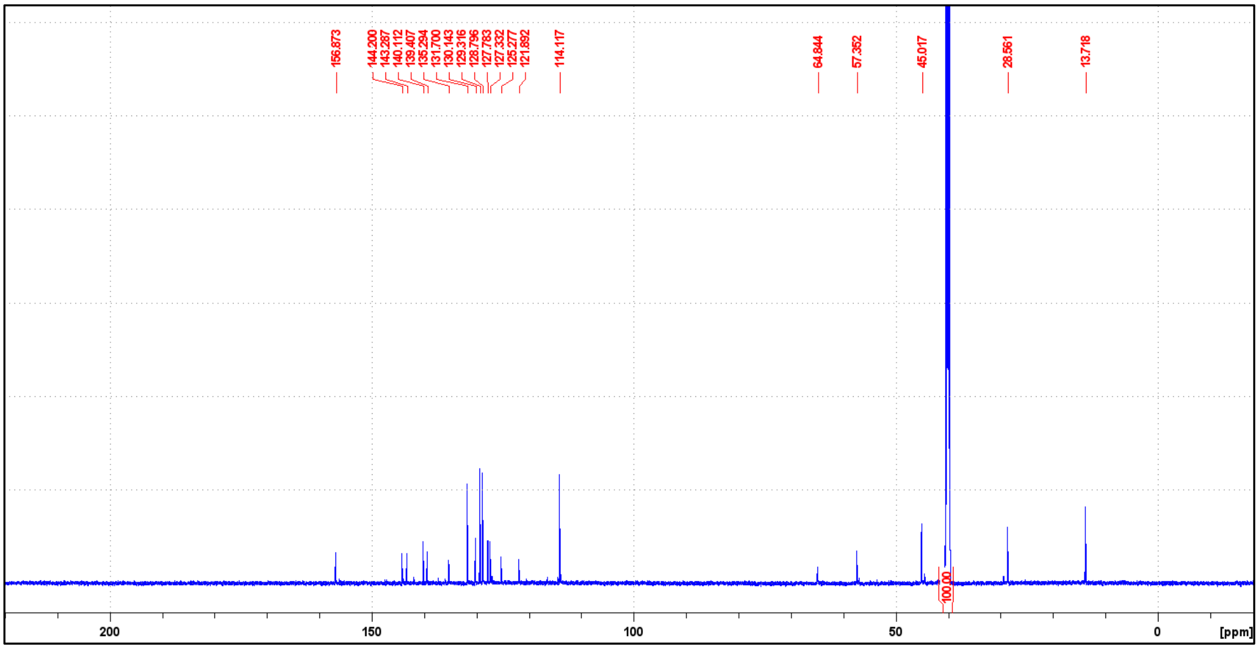

^{13}C-NMR (150 MHz, DMSO-d_6) of compound **1**.

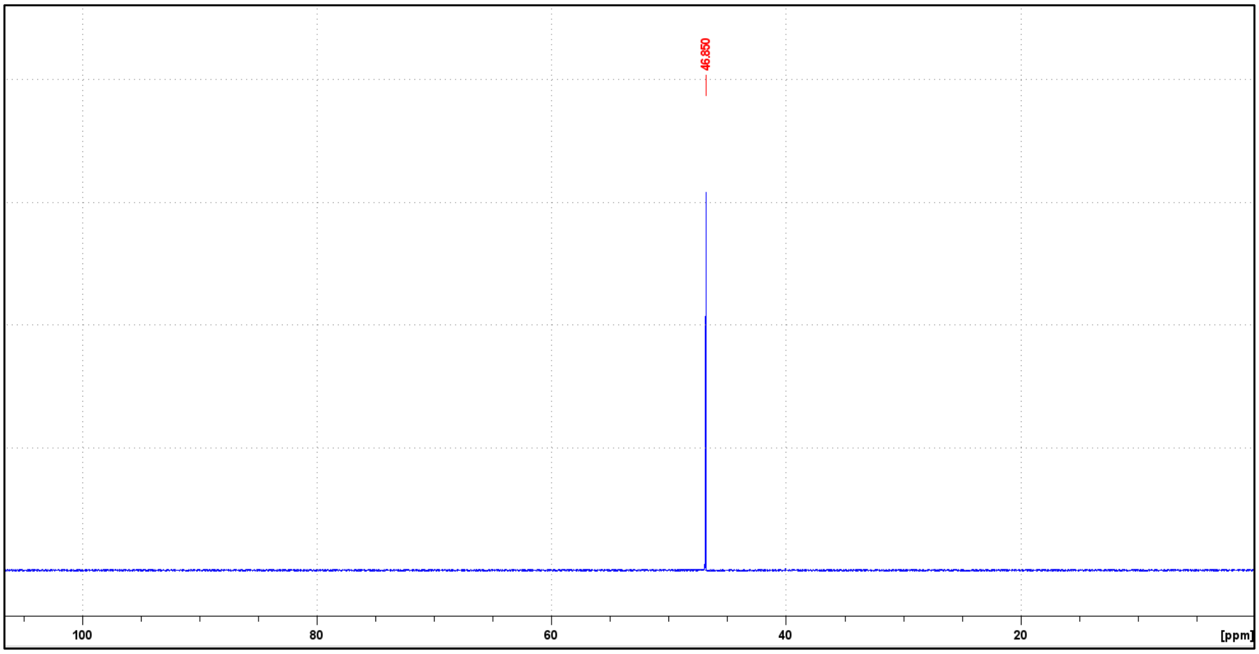

^{19}F-NMR (451 MHz, DMSO-d_6) of compound **1**.

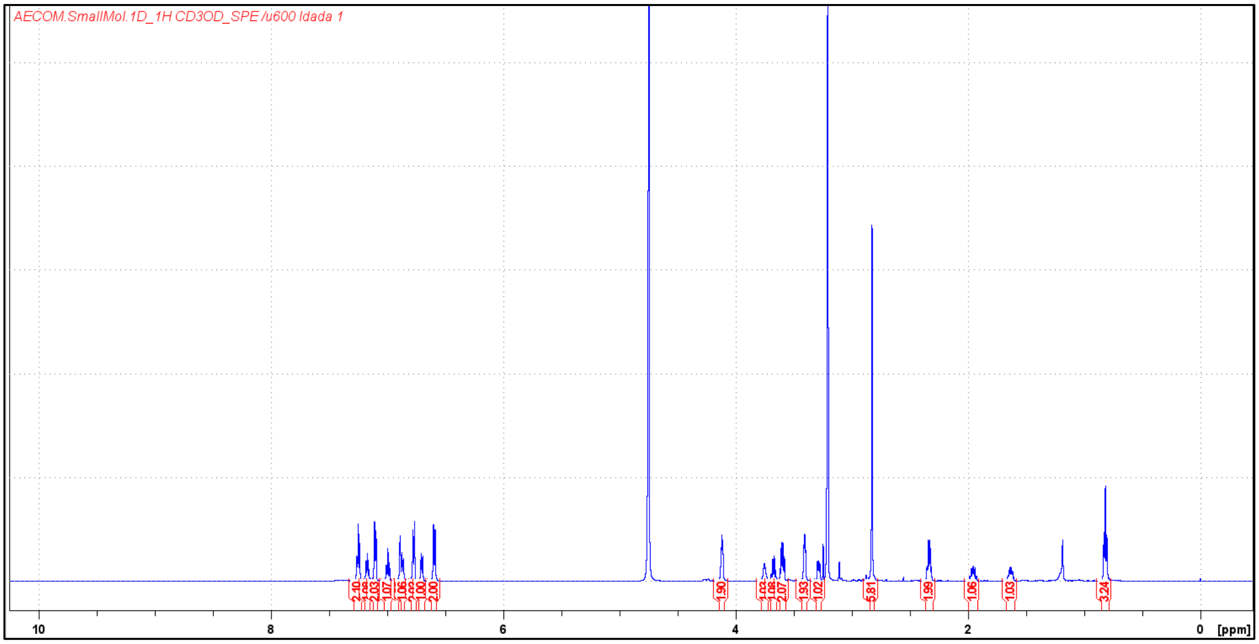

^{1}H-NMR (600 MHz, MeOH-d_4) of compound **4S**.

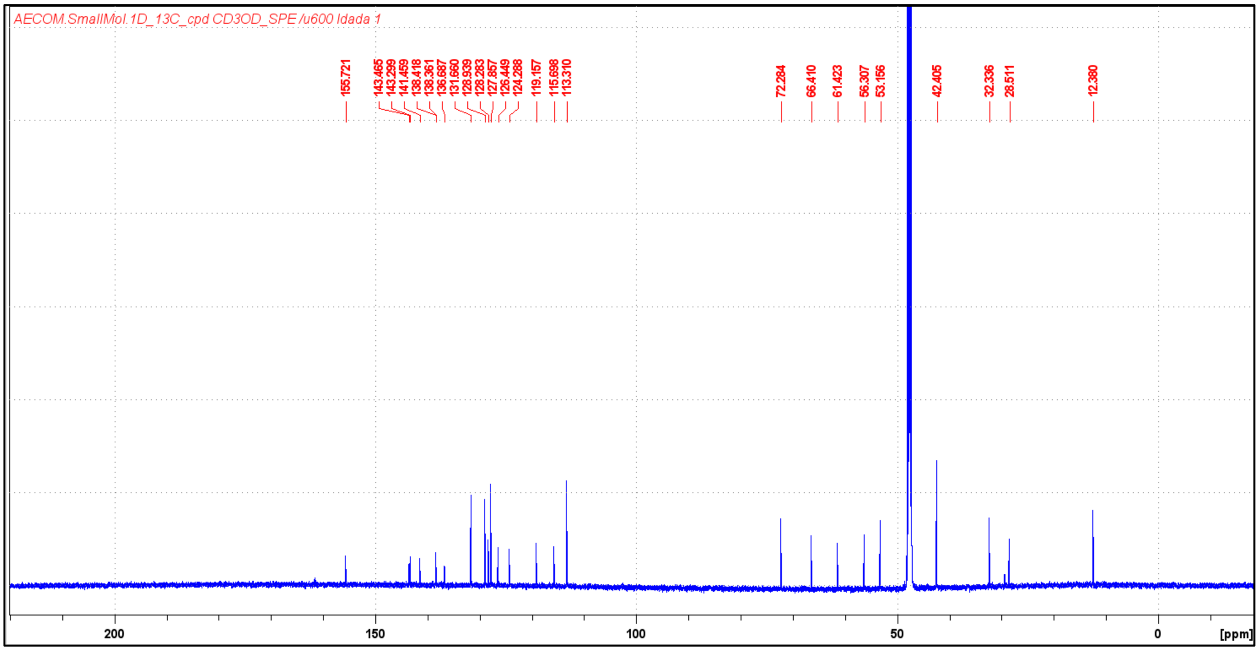

^{13}C-NMR (150 MHz, MeOH-d_4) of compound **4S**.

References

1. Li S, Wu P, Moses JE, Sharpless KB. Multidimensional Sufex Click Chemistry: Sequential Sulfur(VI) Fluoride Exchange Connections of Diverse Modules Launched from an SOF_4 Hub. *Angewandte Chemie International Edition* (2017) 56(11):2903-8. doi: 10.1002/anie.201611048.
